# Empirically Estimated Electron Lifetimes in the Earth's Radiation Belts: Comparison With Theory

**DOI:** 10.1029/2019GL086056

**Published:** 2020-02-07

**Authors:** S. G. Claudepierre, Q. Ma, J. Bortnik, T. P. O'Brien, J. F. Fennell, J. B. Blake

**Affiliations:** ^1^ Space Sciences Department The Aerospace Corporation El Segundo CA USA; ^2^ Department of Atmospheric and Oceanic Sciences UCLA Los Angeles CA USA; ^3^ Center for Space Physics Boston University Boston MA USA

**Keywords:** radiation belt, lifetime, decay, pitch angle diffusion, loss, wave particle interaction

## Abstract

We compute quasilinear diffusion rates due to pitch angle scattering by various mechanisms in the Earth's electron radiation belts. The calculated theoretical lifetimes are compared with observed decay rates, and we find excellent qualitative agreement between the two. The overall structure of the observed lifetime profiles as a function of energy and L is largely due to plasmaspheric hiss and Coulomb scattering. The results also reveal a local minimum in lifetimes in the inner zone at lower energy ( ∼50 keV), attributed to enhanced scattering via ground‐based very low frequency transmitters, and a reduction in lifetimes at higher L and energy ( >1 MeV), attributed to enhanced electromagnetic ion cyclotron wave scattering. In addition, we find significant quantitative disagreement at L<3.5, where the theoretical lifetimes are typically a factor of ∼10 larger than the observed, pointing to an additional loss process that is missing from current models. We discuss potential factors that could contribute to this disagreement.

## Key Points

- Electron scattering by Coulomb collisions, hiss, EMIC, and VLF transmitter waves is demonstrated in observed loss timescales
- The energy‐ L structure of the observed electron lifetimes is in excellent qualitative agreement with quasilinear theory
- Theoretical lifetimes in the inner region ( L<3.5) are much longer than the observed (1,000 vs. 100 days), pointing to a missing loss process

## 1 Introduction

Observations of exponentially decaying fluxes in the Earth's radiation belts suggest that the prevailing particle dynamics are governed by pitch angle diffusion, as described by the modified Fokker‐Planck equation (e.g., Lyons & Thorne, 1973):

(1) $$\frac{\partial f}{\partial t}=\frac{1}{T\sin(2\alpha )}\frac{\partial }{\partial \alpha }\left(D_{\alpha \alpha }T\sin(2\alpha )\frac{\partial f}{\partial \alpha }\right)+S-L.$$

Here, f is the distribution function (phase space density), α is the equatorial pitch angle, $D_{\alpha \alpha }$ is the bounce‐averaged pitch angle diffusion coefficient, T≈1.30−0.56sinα is a term that approximates the pitch angle dependence of the normalized bounce time along a dipole field line, S is an arbitrary source term, and L is an arbitrary loss term (e.g., magnetopause shadowing). The fact that we observe exponential decays suggests that S is small and in what follows we assume S≈0. Similarly, as described in the companion manuscript, very rapid losses like magnetopause shadowing are effectively excluded from our empirical lifetime database, and in what follows we assume L≈0. Under the additional assumption that the solution to equation (1) is separable in α and t, then a solution with time dependence ∼exp(−t/τ) yields an ordinary differential equation (ODE) for the evolution of the angular distribution. The resulting ODE is of a Sturm‐Liouville type, so that the eigenvalues ( =1/τ) are real and ordered, each with a corresponding eigenfunction. The smallest of the eigenvalues, which corresponds to the longest decay timescale τ, dominates the long‐term evolution of the particles, once any transient behavior has subsided (e.g., other eigenmodes).

Within this framework, the second‐order ODE for the angular distribution is usually subject to the boundary conditions that f→0 at $\alpha \leq \alpha_{L}$ and ∂f/∂α→0 at $\alpha =90^{\circ }$, where $\alpha_{L}$ is the loss cone angle. The resulting boundary value problem can be solved analytically only for very simple forms of the diffusion coefficient, $D_{\alpha \alpha }$. For more general forms, standard numerical techniques can be used to obtain the eigenvalues and eigenfunctions (e.g., Albert, 1994). Alternatively, Albert and Shprits (2009) derive an approximate form for the eigenvalue, τ, which can be easily evaluated via:

(2) $$\tau \approx \int_{\alpha_{L}}^{\pi /2}\frac{1}{2D_{\alpha \alpha }\tan\alpha }d\alpha .$$

Throughout this work, we use this approximation for the theoretical particle “lifetime” due to pitch angle diffusion, as is common in other works (e.g., Orlova et al., 2016). (In section 4, we return briefly to this point to discuss the validity of this approximation for τ.) In the companion paper, we have calculated the decay timescales, τ, from observations of exponentially decaying electron fluxes as a function of particle energy and L shell, and we proceed under the assumption that such decays are representative of pitch angle diffusion in the lowest order eigenmode of the diffusion operator defined by equation (1). The goal of the present paper is to compare the observed lifetimes with theoretical estimates due to quasilinear pitch angle diffusion by various scattering processes, given via equation (2). Such comparisons constrain and inform our understanding of the physics by evaluating how well our current wave models capture the relevant loss processes. For example, when theoretical lifetimes do not match observed decay timescales, it can suggest that new and/or additional physical processes that were not previously known or believed to be important may be operating. In addition, ring current and radiation belt models often require electron loss timescales as one of the model parameters (e.g., Chen et al., 2015;Ozeke et al., 2017), and a number of studies have shown that the model results are highly sensitive to the assumed lifetimes (Aseev et al., 2019; Ganushkina et al., 2019).

## 2 Data and Methods

**Figure 1:**
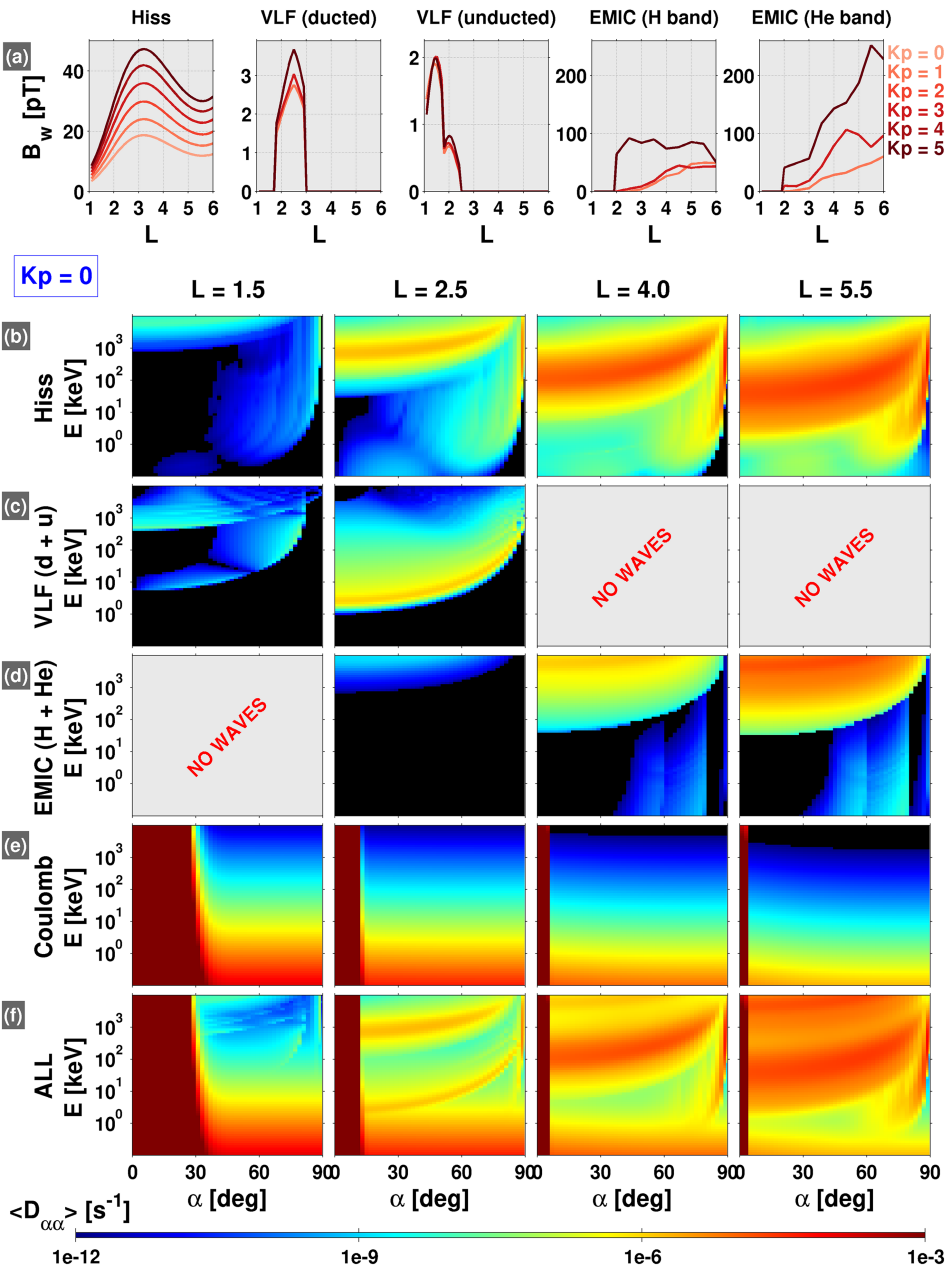
(a) Wave amplitudes assumed in the diffusion coefficient calculations, as a function of L for all Kp. (b–f) Bounce‐averaged pitch angle diffusion coefficients, $<D_{\alpha \alpha }>$, as a function of equatorial pitch angle and energy due to the indicated scattering mechanism (rows) at various L (columns), for Kp = 0.

To calculate the theoretical lifetimes, we must specify quasilinear diffusion coefficients due to waves that are known to exist and be important for pitch angle scattering in the radiation belt region. Since we will compare the theoretical lifetimes with mean empirical lifetimes obtained from a statistical database of 5 years of observations, and thus a wide range of geomagnetic activities and wave environments, we use statistical wave models to specify the diffusion coefficients. For example, the first panel in Figure 1a shows L profiles of hiss wave amplitudes obtained from the empirical model of Spasojevic et al. (2015). The statistical wave amplitudes are provided as a function of L, magnetic local time (MLT), and geomagnetic activity parameterized by the Kp index, 0–5. The profiles in Figure 1a are averaged over all MLT.

The assumed statistical wave amplitudes, such as those shown for hiss in Figure 1a, are used to compute bounce‐averaged, quasilinear, electron pitch angle diffusion coefficients using the “Full Diffusion Code” (Ni et al., 2008). There are several aspects of these calculations that are common to all of the wave modes considered. The geomagnetic field is assumed to be dipolar, the latitudinal range for the resonant interactions is assumed to be $\pm 45^{\circ }$ around the magnetic equator, and resonant harmonics from −10 to +10 are considered. At low L shells, the waves are also confined below the magnetic latitude where the magnetic field line reaches 800‐km altitude from the Earth's surface. The plasma density is specified using the empirical plasmaspheric density model of Ozhogin et al. (2012) at L<4, and the plasmaspheric density model of Sheeley et al. (2001) is used at L>4. The calculations are carried out from L=1−6 in 0.1 L‐width bins, from 0.1 keV to 10 MeV in 71 logarithmically spaced energy channels, and for equatorial pitch angles from 1° to 89.5° with Δα=2° . The diffusion coefficients are also drift averaged, since we are concerned with multiday electron dynamics where drift‐timescale effects are unimportant. We note that the use of daily‐averaged fluxes in our empirical lifetime database ensures that we are considering drift‐bounce‐averaged fluxes, appropriate for comparisons with theoretical lifetimes calculated from drift‐bounce‐averaged diffusion coefficients.

Calculating the diffusion coefficients also requires several additional assumptions on the wave parameters (e.g., wave frequency spectrum and wave normal angle spectrum). For hiss waves, we use the statistical frequency spectrum of Li et al. (2015) and extrapolate the spectrum from 4 to 7 kHz as an approximate means of incorporating lightning‐generated whistler waves (this is discussed further in section 4). The wave normal angle spectrum is from Ni et al. (2013), which is specified as quasi‐field aligned at the magnetic equator, progressing to highly oblique at 45° latitude. Figure 1b shows the bounce‐averaged diffusion coefficients computed for these assumed hiss wave parameters at four different L values. The region of enhanced scattering at higher energy that occurs over a wide range of pitch angles is mostly due to the cyclotron resonances, whereas the region of enhanced scattering that is narrow in pitch angle near 90° is due to the Landau resonance.

In addition to hiss waves, we also calculate the scattering rates for very low frequency (VLF) transmitter waves, electromagnetic ion cyclotron (EMIC) waves, and Coulomb collisions. The statistical parameters assumed for these scattering processes are described in greater detail in the supporting information. Figure 1a shows the statistical wave amplitudes for both ducted and unducted VLF transmitter waves and both proton (H) and Helium (He) band EMIC waves. We note that the VLF waves are limited to L<3 and the EMIC waves to L>2 based on the best‐available statistical wave databases (see supporting information). The bounce‐averaged diffusion coefficients computed for the assumed wave parameters are shown in Figure 1c for VLF transmitter waves, where $D_{\alpha \alpha }$ = $D_{\alpha \alpha }^{ducted}$ + $D_{\alpha \alpha }^{unducted}$, and in Figure 1d for EMIC waves, where $D_{\alpha \alpha }$ = $D_{\alpha \alpha }^{H}$ + $D_{\alpha \alpha }^{He}$. Figure 1e shows the bounce‐averaged diffusion coefficients computed for Coulomb scattering, and panel (f) shows the combined scattering rates due to all of the relevant mechanisms described, where $D_{\alpha \alpha }$ is defined as the summation of all of the constituent diffusion coefficients. Further details regarding the diffusion coefficient calculations are provided in the supporting information.

## 3 Results

**Figure 2:**
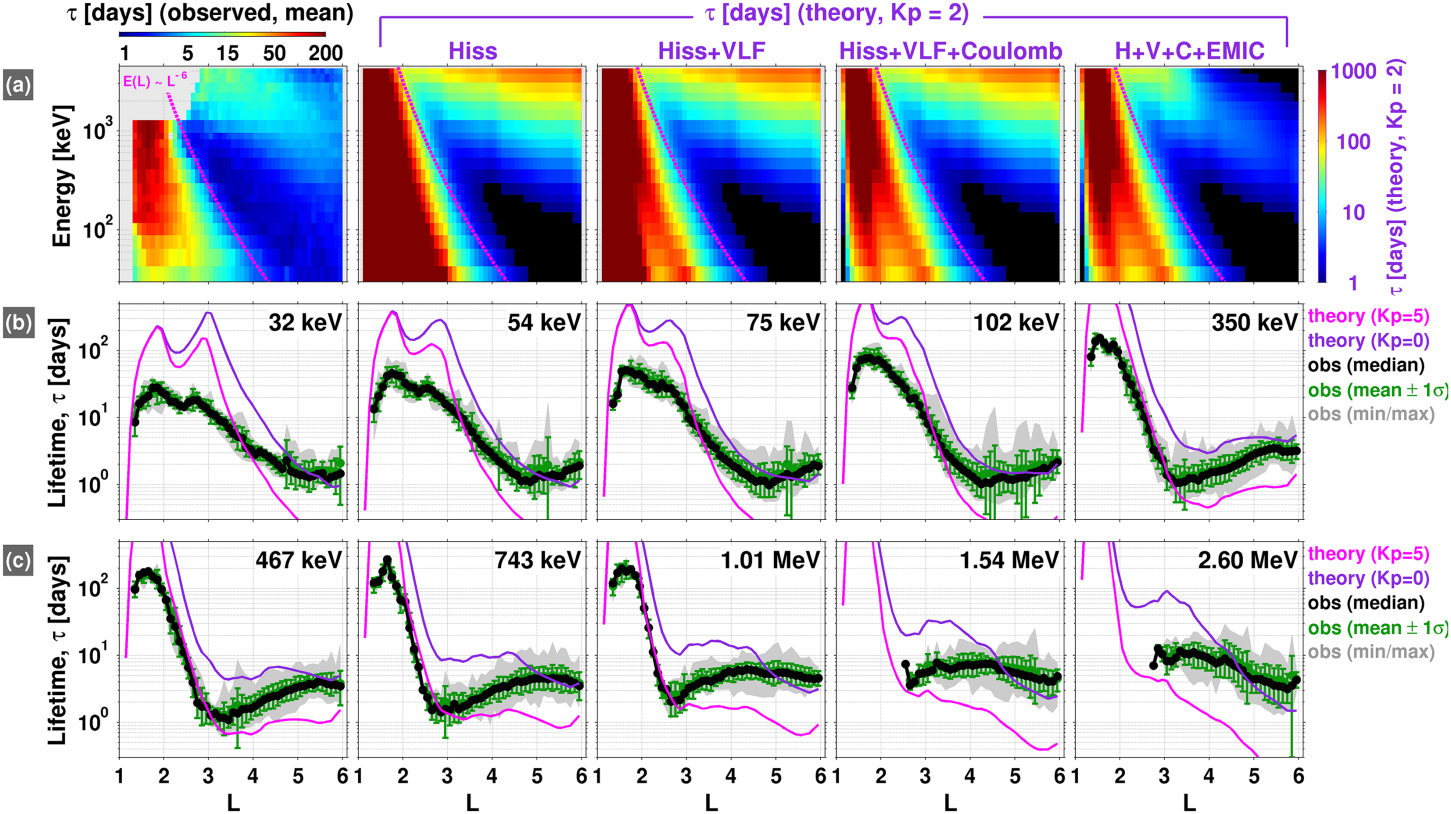
(a) Observed mean lifetimes (first panel) along with the theoretical predictions for pitch angle diffusion due the indicated scattering mechanism(s). Note that the color scale is different for the observed and theoretical lifetimes (1–200 days vs. 1–1,000 days, respectively). (b, c) Same as panel (a) but presented in a line plot format, where the mean observed lifetimes are shown in green with 1σ error bars, along with the median (black), and a shaded region (grey) indicating the minimum and maximum values at each L. The theoretical lifetimes in these two rows (pink and purple) are obtained from the $D_{\alpha \alpha }$ where all of the scattering mechanisms are combined: hiss, VLF, Coulomb, and EMIC (e.g., the last panel in row (a)).

Figure 2a compares the empirical lifetimes obtained in the companion paper (first panel) with the theoretical lifetimes computed via equation (2) for various combinations of the diffusion coefficients. The “Hiss” (second) panel shows the theoretical lifetimes calculated only for scattering by plasmaspheric hiss for Kp=2, which is roughly the mean Kp for all of the decay intervals identified (not shown here). Comparing the first two panels reveals a good qualitative agreement between the lifetime profiles, with several common features: The longest lifetimes are found in the inner zone; a slot region where the lifetimes drop precipitously to their minimum values; and the outer zone where the lifetimes increase again, but not nearly to the levels found in the inner zone. It is clear that the rapid scattering rates due to hiss waves in the intermediate L and energy ranges carve out the slot region. This central role that hiss waves play in forming the slot is a well‐known result (e.g., Lyons & Thorne, 1973), but we emphasize that it is clearly reproduced here in the empirical lifetime estimates. Note that the scaling of the minimum energy for cyclotron resonance with whistler mode waves is roughly $∼L^{-6}$ for the plasmaspheric density model and dipolar magnetic field used here (Ma et al., 2016; Mourenas et al., 2012, 2017) and this profile is shown in each of the panels in row (a). We note that the reason for the gap in the observed lifetimes at energies >1 MeV and L<3 is that there have not been injections of >1.5‐MeV electrons into the inner zone at detectable levels during the Van Allen Probes era (Claudepierre et al., 2019; Fennell et al., 2015).

The “Hiss+VLF” panel in Figure 2a shows the theoretical lifetimes due to both hiss and VLF transmitter waves. Comparing the Hiss and Hiss+VLF panels reveals that the inclusion of the VLF transmitter waves has a significant impact on the lifetimes in the inner zone, particularly at energies less than ∼300 keV. Overall, the theoretical lifetimes in the inner zone are reduced when the transmitter waves are included. In addition, a local minimum is produced in the lifetimes near L=2.5 at the lowest energies shown, 30–300 keV, due to the highly localized transmitter waves. Note that the location of this local minimum moves to lower L as energy increases, consistent with expectations from the cyclotron resonance condition. We emphasize that there is some evidence of a corresponding local minimum in the observed lifetimes (first panel) in roughly the same L and energy range. We return to this point below.

The “Hiss+VLF+Coulomb” panel in Figure 2a shows the theoretical lifetimes due to the combined effects of hiss, VLF transmitter waves, and Coulomb scattering. The influence of Coulomb scattering on the inner zone lifetimes is clear, with a significant reduction in the lifetimes when compared with the Hiss+VLF panel. This is to be expected, as many authors have shown that Coulomb scattering contributes significantly to radiation belt electron loss at L<2.5 and is the dominant scattering mechanism at L<1.5 (e.g., Abel & Thorne, 1998). Note also that the inclusion of Coulomb scattering greatly reduces the lifetimes very close to the earth ( L<1.3) and produces a local maximum near L=1.5 (relative to the profiles in the Hiss+VLF panel), which is seen in the observed lifetimes as well. The inclusion of Coulomb scattering also more clearly reveals the local minimum in lifetime due to VLF transmitter waves at ∼30 keV near L=2.5.

The final panel in Figure 2a incorporates all of the scattering mechanisms considered in this manuscript—hiss, VLF, Coulomb, and EMIC—which constitute the majority of the relevant processes for pitch angle scattering of radiation belt electrons. When compared with the Hiss+VLF+Coulomb (fourth) panel, we see a dramatic reduction in the theoretical lifetimes at the highest energies and L shells, consistent with expectations for EMIC wave scattering of electrons. Note that there is evidence of this reduction in the observed lifetimes as well (first panel).

Figures 2b and 2c display the same data as row (a), but in a line plot format as a function of L at fixed energies. The theoretical lifetimes computed from the diffusion coefficient that incorporates all of the scattering mechanisms (Hiss+VLF+Coulomb+EMIC; e.g., Figure 2a, last panel) are shown in purple for Kp=0 and in pink for Kp=5, for reference. In this regard, we note that while the observed decays, from which the empirical lifetimes are computed, occur over a range of magnetic activity levels, the average Kp is generally low during the decay intervals ( ∼2 or less; not shown here). However, it is difficult to organize the empirical lifetimes on activity level, since the decay intervals are at least 5 days long (and often longer—see companion manuscript), over which time a wide range of activity levels can be observed. Thus, assigning a single Kp value to an entire decay interval is somewhat arbitrary. Similarly, we do not sort the observed decay timescales with respect to the plasmapause location. It is difficult to assign an “inside” or “outside” of the plasmasphere designation to an individual decay event, since the plasmapause can move across the fixed L bin during the decay interval. The majority of the observed decays at L<5 occur primarily inside of the plasmasphere.

## 4 Discussion

Overall, Figure 2 demonstrates that there is good qualitative agreement between the observed lifetimes and those predicted theoretically for quasilinear scattering via the various mechanisms. Comparing the final panel in row (a) with the empirical estimates in the first panel, we see that the morphological structure of the observed lifetimes in energy and L is well predicted by the theory presented. The impact of each of the four scattering mechanisms considered is readily apparent in the observed lifetimes: the short lifetimes in the slot region primarily due to hiss waves; the reduction in inner zone lifetimes with a peak near L=1.5 due to Coulomb scattering; the local minimum in lifetimes between L=2−3 due to VLF transmitter waves; and the reduction in lifetimes at high L and energy due to EMIC waves.

However, Figure 2 also demonstrates that there is significant quantitative disagreement between the observed and theoretical lifetimes. This is particularly true in the inner regions ( L≲3.5), where the theoretical lifetimes (for Kp=0) are larger than the observed by at least a factor of 5 and often by an order of magnitude or more. We note that the theoretical lifetime calculations presented here are largely consistent with prior work using similar techniques (Abel & Thorne, 1998; Albert, 2000; Meredith et al., 2006; Ripoll et al., 2017). Similarly, as detailed in the companion manuscript, our empirical lifetime estimates are in good agreement with prior estimates, when and where such estimates are available (Benck et al., 2010; West et al., 1981; Vampola, 1971). When differences are noted with prior works (either theoretical or empirical, separately), they are typically on the order of a factor of 2, which cannot explain the order‐of‐magnitude differences between theory and observations found here.

### 4.1 Discrepancies Between Observed and Theoretical Lifetimes

We now discuss several possibilities that could explain such differences in the inner regions ( L≲3.5). Perhaps the most important missing piece from our theoretical calculations is the ad hoc incorporation of lightning‐generated whistler waves. The Li et al. (2015) hiss spectrum that is used also includes lightning‐generated waves up to 4 kHz at L≲3.5 (Agapitov et al., 2014; Meredith et al., 2007; Spasojevic et al., 2015), which we extrapolate to 7 kHz as an approximate way to account for lightning‐generated whistlers in our calculations. We also note recent work that demonstrates that wave intensity from lightning‐generated whistlers can reach substantially higher frequencies ( ∼12 kHz) at L<3 (Záhlava et al., 2019). Future work will incorporate lightning‐generated whistler waves in a more rigorous fashion.

There may also be shortcomings and areas for improvement in our assumptions surrounding the VLF transmitter waves. For example, there is considerable uncertainty in the wave normal angle spectrum for VLF transmitter waves (Ma et al., 2017). In our calculations, we assume that the VLF transmitter waves at L≲1.7 are unducted and have large wave normal angles. To this end, we performed a test calculation where we assumed all VLF transmitter waves were field aligned from L=1−3. The results (not shown here) did show a reduction in the lifetimes, but only by a factor of 2–3, and we note that this assumption of all waves being field aligned is a gross over‐simplification. An additional area for improvement may be the method by which Ma et al. (2017) represented the wave frequency spectrum, where Gaussian functions were fit on the central frequency only, noting that measurable (but lower) wave power is present at other frequencies. It is possible that including this additional wave power could reduce the lifetimes. For example, very recent works (Meredith et al., 2019; Ross et al., 2019) fit a Gaussian function to each VLF transmitter station, and the resulting lifetimes are somewhat lower than those calculated here, but again only by a factor of 2–3.

There are also additional processes in the inner region that can scatter radiation belt electrons in pitch angle. For example, magnetosonic wave activity is known to occur down to at least L=2 (Ma et al., 2016). While these waves themselves do not cause significant precipitation loss of energetic electrons inside the plasmasphere, they can contribute to losses by acting in concert with other waves (e.g., hiss). In addition, recent work has discovered that He‐band EMIC waves, the dominant band observed in the inner magnetosphere, are frequently observed below L=4, down to at least L=2 (Gamayunov et al., 2018). However, the distribution and characteristics of these low‐ L EMIC waves have yet to be quantified so that they can be incorporated into radiation belt modeling. We have also not considered a number of known aspects of plasmaspheric hiss, such as oblique hiss (e.g., Hartley et al., 2018) and low‐frequency hiss (e.g., Ni et al., 2014), which could influence our lifetime calculations at low L. Coulomb energy drag (ionization energy loss), whereby electrons lose energy when they ionize atoms in the ambient neutral atmosphere and ionospheric plasma, may also be important to consider in future work.

The agreement between the theory and observations is much better in the outer L regions, L≳3.5, where the theoretical lifetimes for Kp=0 are generally within a factor of 3 of the observed (e.g., Figures 2b and 2c). However, at the highest energies shown (e.g., >500 keV), we note that the theoretical profiles generally decrease faster in L than the observed profiles. This may suggest that the EMIC wave scattering is too strong in our calculations, which is discussed in the supporting information. We also note that we have not included chorus‐wave scattering, which will contribute to losses outside of the plasmasphere. Both of these mechanisms will be considered and refined in future work.

We have investigated a number of other factors that could potentially lead to discrepancies between the observations and theory, though none has provided a satisfactory explanation. First, as noted above, equation (2) represents an approximation to the true theoretical lifetime. We have carried out the full, exact calculation of τ via a shooting method (e.g., Albert, 1994). While we do find that the exact lifetimes are somewhat lower than the approximated, these differences are not larger than a factor of ∼2 (and often they agree quite well) and thus not sufficient to explain order‐of‐magnitude discrepancies in the inner region. Another potential factor is the inclusion of higher order cyclotron resonances, beyond the ±10 that we have considered. For example, Albert (1994) included up to ±100 and found a reduction in theoretical lifetimes by a factor of 2–3, which again is unlikely to explain order‐of‐magnitude differences (see also Meredith et al., 2006). Note that both of these factors will affect the theoretical lifetime estimates everywhere, whereas the most significant discrepancies are at lower L and thus likely not due to some systematic effect. A third potential factor could be an artifact related to the data processing and automated algorithm that are used to obtain the empirical lifetime estimates. However, in the companion paper, we demonstrate good quantitative agreement with previous empirical estimates, lending confidence that our empirical estimates are accurate. When differences are noted with the previous works, they are typically on the order of a factor of 2, and not an order of magnitude. While it is plausible that a combination of some of the effects described above could account for the discrepancy, we feel that it is unlikely that these uncertainties would all conspire together in the same direction to explain the discrepancy between theory and observation presented here.

Finally, we note that the empirical lifetimes could potentially be influenced by a source (e.g., inward radial transport from higher L) and thus may not always be representative of the true, underlying decay timescale (see companion paper). However, our inability to account for this in the empirical lifetime database cannot explain the discrepancies noted, since if a source is present, it will act to artificially increase the lifetime estimate over its true value (i.e., $\tau_{true}\leq \tau_{observed}$), but $\tau_{observed}<<\tau_{theory}$ at lower L (i.e., the noted discrepancy would be even larger with $\tau_{true}$). At higher L≳5, radial diffusion can act as both a source and a loss (e.g., outward transport to the magnetopause in the presence of a negative gradient in phase space density), further complicating the picture.

### 4.2 Bifurcated Inner Belt at E<100 keV

We now focus on the L profiles of the lifetimes at the lowest energies, 32–75 keV, in Figure 2b. As noted above, the electron interactions with VLF transmitter waves lead to a local minimum in the theoretical profiles between L = 2–3, the location of which clearly moves inward as energy increases. We see evidence of this local minimum in the observed lifetimes as well, in nearly the same L region and with the same energy‐dependent location, though the minimum is more pronounced in the theoretical lifetimes. This latter effect may be related to source processes bringing freshly injected electrons into the low L region (e.g., Turner et al., 2017) and the fact that our empirical lifetimes are upper bounds, as noted above. As further evidence to support the claim that the VLF transmitter influence is reflected in the observed lifetimes, we demonstrate that the effect is notable in the raw flux measurements as well.

**Figure 3:**
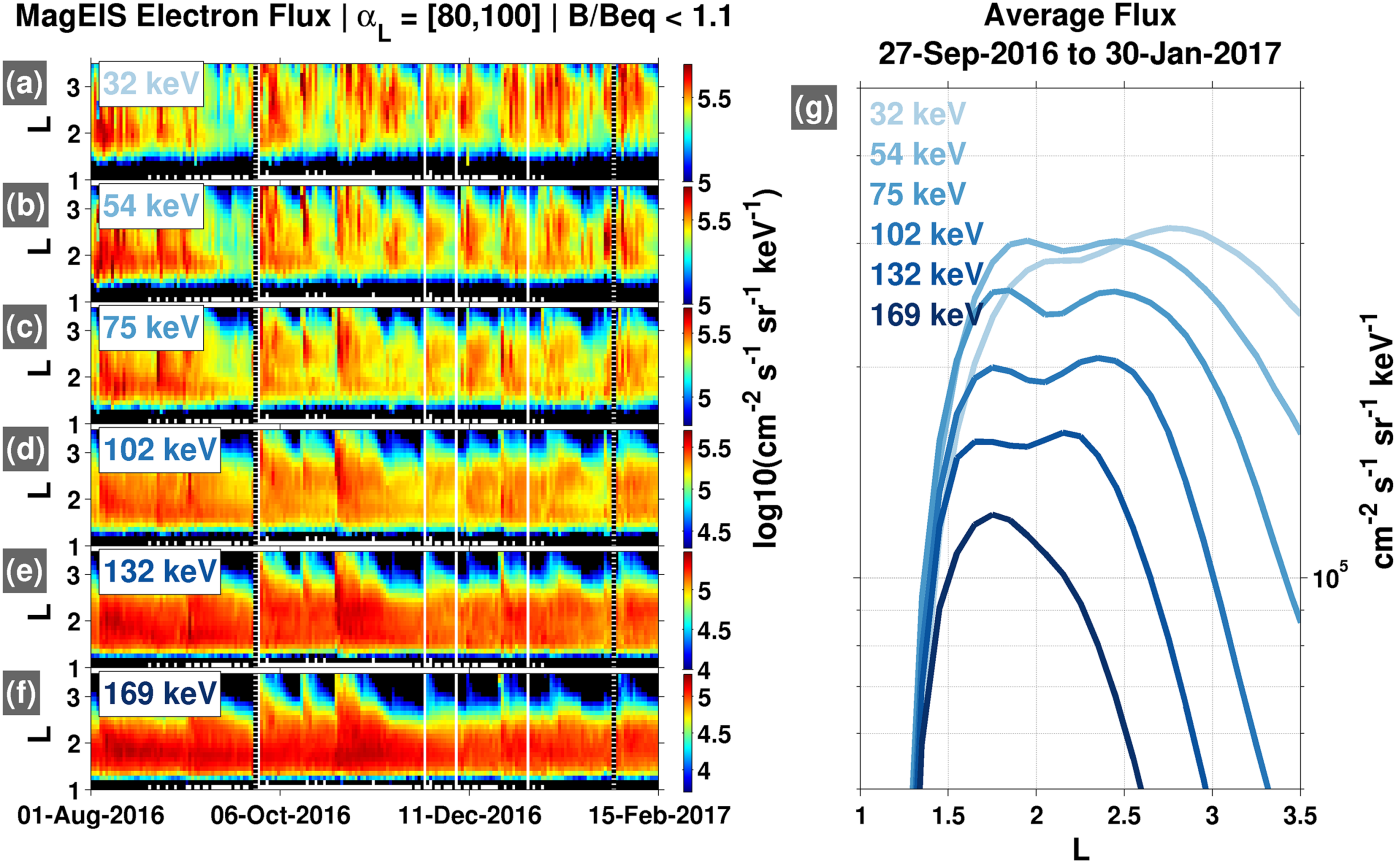
Ground‐based VLF transmitter influence on electron lifetimes manifested in flux measurements, where a local minimum in flux is observed between L = 2–3 at low energy (30–100 keV), producing a bifurcated inner zone. (a–f) Low energy fluxes in L versus time format. (g) Fluxes averaged over the indicated time interval.

Figures 3a–3f show L versus time profiles of electron flux from ∼30 to 200 keV for a ∼6‐month interval. In panels (a)–(d), we see evidence of a local minimum in the flux between L = 2–3, in precisely the same location predicted from our theoretical considerations of the VLF transmitter wave influence. Following electron injections/enhancements in the L<3 region, the fluxes decay rapidly near L=2 when compared with the flux evolution at adjacent (higher and lower) L shells, perhaps most clearly seen in the 75 keV panel. Moreover, the location in L of this local minimum in flux is energy dependent, moving to lower L as energy increases, again consistent with our findings from the lifetime calculations. This fact is made clear in Figure 3g, where L profiles of time‐averaged fluxes are presented, with the location of the local flux minimum moving earthward with increasing energy, and disappearing entirely at E≳ 160 keV, consistent with the theoretical estimates shown in Figure 2. While the local minimum is small in terms of the relative flux levels, the totality of the evidence presented demonstrates that the VLF transmitter waves produce a bifurcated, two‐belt inner zone morphology, with a local minimum in flux between L=2–3.

## 5 Summary

This manuscript presents a comprehensive comparison of observed and theoretical radiation belt electron lifetimes, the first such analysis made with observations from a near‐equatorial, high‐altitude observational platform spanning nearly half of a solar cycle. The use of empirical lifetime estimates obtained from a statistical database of radiation belt decay events reveals the influence of a multitude of scattering processes on the radiation belts (hiss, VLF transmitter, EMIC, and Coulomb scattering). Our findings are consistent with recent work that has linked morphological features in radiation belt observations to the action of plasmaspheric hiss wave‐driven pitch angle diffusion (e.g., the “reversed” or “bump‐on‐tail” energy spectrum and the “wave‐like” or “S‐shaped” spectrum; Ma et al., 2016; Reeves et al., 2016; Ripoll et al., 2016; Zhao et al., 2019). However, when compared with the theoretical lifetimes due to pitch angle diffusion, it is demonstrated that hiss waves alone cannot explain all of the observed structure; EMIC waves are demonstrated to be important at higher energy ( ≳1 MeV) and higher L, while Coulomb and VLF transmitter scattering are demonstrated to be important at lower L. We emphasize that an anthropogenic (human) influence on the inner radiation belt is demonstrated clearly in the observations, where ground‐based VLF transmitter waves lead to enhanced scattering of ∼50‐keV electrons in a narrow L region. While all of these effects have been previously reported, the high‐quality measurements used here allow us to make truly quantitative assessments of how well our current, best‐available empirical wave models capture the effects of quasilinear pitch angle diffusion in the radiation belts. The analysis reveals that the theoretical lifetimes calculated are much longer than the observed values in the inner regions ( L≲3.5) and indicate that something may be missing from our current understanding of the relevant scattering processes there. Of the known mechanisms, only lightning‐generated whistlers are not rigorously accounted for in our analysis, though they are included in an ad hoc manner. Future work will properly account for these and other waves (e.g., magnetosonic waves) and processes (e.g., Coulomb drag), one of which may prove to be the “missing” piece that brings the theory into closer alignment with the observations.

## Supporting information



Supporting Information SI
