# Supplementary material for: Empirically Estimated Electron Lifetimes in the Earth's Radiation Belts: Comparison With Theory
Source: Geophys Res Lett. 2020 Feb 7;47(3):e2019GL086056. doi: 10.1029/2019GL086056 (PMC7375138; doi:10.1029/2019GL086056)
Supplement: Supplementary file 1 — Supporting Information SI [file GRL-47-e2019GL086056-s001.pdf]

# Supporting Information for "Empirically estimated electron lifetimes in the Earth's radiation belts: 2. Comparison with theory"

S. G. Claudepierre[1,2], Q. Ma[2,3], J. Bortnik[2], T. P. O'Brien[1], J. F. Fennell[1],

and J. B. Blake[1]

[1]Space Sciences Department, The Aerospace Corporation, El Segundo, California, USA

[2]Department of Atmospheric and Oceanic Sciences, UCLA, Los Angeles, California, USA

[3]Center for Space Physics, Boston University, Boston, Massachusetts, USA

## Contents of this file

1. Text S1

## Introduction

## Text S1.

The following information is provided in support of the main manuscript and gives additional detail regarding the diffusion coefficient calculations.

Radio waves from ground-based, high-powered very-low frequency (VLF) transmitters can escape out of the Earth-ionosphere waveguide and into near-Earth space where they propagate in the whistler mode. These waves are known to interact with and scatter

---

electrons out of the radiation belts and into the Earth's upper atmosphere (Inan et al., 1984; Koons et al., 1981). Thus, they must be included in our considerations and we incorporate them following Ma, Mourenas, Li, Artemyev, and Thorne (2017). Ma et al. (2017) carried out these calculations from $L = 1 - 3$ in $0.1L$-width bins for three $Kp$ bins (0-2, 2-4, and >4) over the frequency range 10-30 kHz. The wave amplitudes used in this study have been averaged over all MLT. The assumed wave normal angle spectrum for the ducted waves, which are assumed to only be present at $L > 1.7$, is quasi-field aligned, while the unducted waves are assumed to be oblique with wave normal angles $\sim$60°-70°. As in Ma et al. (2017), the wave frequency spectrum is fit with a Gaussian to capture the wave power from the most intense ground transmitter at frequency of 20 kHz.

Electromagnetic ion cyclotron (EMIC) waves can resonantly interact with electrons at radiation belt energies, primarily resulting in pitch angle scattering. For our calculations, the assumed wave frequency and amplitude spectra are taken from Zhang et al. (2016) and both proton (H) and Helium (He) band waves are incorporated. We note that the quiet, moderate, and disturbed AL* bins used by Zhang et al. (2016) to organize the wave activity have been recast into three $Kp$ bins (0-1, 2-3, and 4+, respectively). The wave normal angle spectrum is obtained from Ni et al. (2015) (their "latitudinally varying model") where the waves are quasi-field aligned at the magnetic equator and increase in obliquity towards higher latitudes (where wave normal angles $\sim$45°). Incorporating EMIC waves also requires an additional assumption on the ion composition and we assume a 70/20/10% proton/Helium/Oxygen plasma, a conventional assumption used in

many prior works (e.g., Meredith et al., 2003). We note that the plasma composition in individual events can be somewhat different from this partitioning, which we have explored but find that using different ratios only affects the overall lifetimes by a factor ∼2 (see below).

In general, EMIC-wave scattering of radiation belt electrons is difficult to include in quasilinear modeling calculations like those presented here, due to their sporadic, bursty nature and the fact that their statistical distribution and wave properties are not well-parameterized. For example, significant EMIC wave scattering at low $Kp$ may be somewhat unrealistic and assuming that they act over multi-day time intervals may affect our results (though we do note that we account for the EMIC wave occurrence rates, as reported by Zhang et al. (2016)). Moreover, the EMIC wave minimum resonant energy is sensitive to both the assumed ion composition and the cold plasma approximation with the assumption that the wave power is close to the upper helium (or hydrogen) band. Under the assumptions used in the present study, the minimum resonant energy is below 1 MeV and may lead to artificially lower theoretical lifetimes at higher $L$ for energies between 500 keV and ∼2 MeV (below 500 keV, hiss scattering dominates over EMIC at higher $L$). With regard to the ion composition, we have performed a test calculation to explore the sensitivity of the lifetimes on the assumed composition ratios. In this calculation, we assumed a 90/5/5% proton/Helium/Oxygen plasma, which is more typical of some observational event studies (e.g., Lee & Angelopoulos, 2014; Kersten et al., 2014; Blum et al., 2019; Qin et al., 2019). We find lower lifetimes at higher energies and $L$, but only

by a factor of ∼2. In summary, in spite of the challenges associated with incorporating EMIC waves into quasilinear scattering calculations, it is clear from the results presented that EMIC waves must be included in order to bring the theoretical calculations more in line with the observations.

Coulomb scattering arises when radiation belt electrons are absorbed by the Earth's upper atmosphere due to Coulomb collisions. This is an important scattering mechanism, particularly close to the Earth in the inner zone, but also for electrons at high-altitudes with low-alitude mirror points. We follow the methodology of Abel and Thorne (1998) to incorporate Coulomb scattering into our calculations, obtaining the atmospheric neutral species ($N_2$, $O_2$, Ar, He, O, H, N) from the MSIS90 empirical model (Hedin, 1991) and the charged species ($e^-$, $NO^+$, $O^+$, $O_2^+$, $H^+$, $He^+$, $N^+$) from the IRI2016 model (Bilitza et al., 2017).

## References

Abel, B., & Thorne, R. M. (1998, February). Electron scattering loss in Earth's inner magnetosphere 1. Dominant physical processes. *J. Geophys. Res.*, *103*, 2385-2396. doi: 10.1029/97JA02919

Bilitza, D., Altadill, D., Truhlik, V., Shubin, V., Galkin, I., Reinisch, B., & Huang, X. (2017). International reference ionosphere 2016: From ionospheric climate to real-time weather predictions. *Space Weather*, *15*(2), 418-429. doi: 10.1002/2016SW001593

Blum, L., Artemyev, A., Agapitov, O., Mourenas, D., Boardsen, S., & Schiller, Q. (2019). Emic wave-driven bounce resonance scattering of energetic electrons in the inner magnetosphere. *J. Geophys. Res.*, *124*(4), 2484-2496. doi: 10.1029/2018JA026427

Hedin, A. E. (1991). Extension of the msis thermosphere model into the middle and lower atmosphere. *J. Geophys. Res.*, *96*(A2), 1159-1172. doi: 10.1029/90JA02125

Inan, U. S., Chang, H. C., & Helliwell, R. A. (1984). Electron precipitation zones around major ground-based vlf signal sources. *J. Geophys. Res.*, *89*(A5), 2891-2906. doi: 10.1029/JA089iA05p02891

Kersten, T., Horne, R. B., Glauert, S. A., Meredith, N. P., Fraser, B. J., & Grew, R. S. (2014, November). Electron losses from the radiation belts caused by EMIC waves. *J. Geophys. Res.*, *119*, 8820-8837. doi: 10.1002/2014JA020366

Koons, H. C., Edgar, B. C., & Vampola, A. L. (1981). Precipitation of inner zone electrons by whistler mode waves from the vlf transmitters ums and nwc. *J. Geophys. Res.*, *86*(A2), 640-648. doi: 10.1029/JA086iA02p00640

Lee, J. H., & Angelopoulos, V. (2014). Observations and modeling of emic wave properties in the presence of multiple ion species as function of magnetic local time. *J. Geophys. Res.*, *119*(11), 8942-8970. doi: 10.1002/2014JA020469

Ma, Q., Mourenas, D., Li, W., Artemyev, A., & Thorne, R. M. (2017). Vlf waves from ground-based transmitters observed by the van allen probes: Statistical model and effects on plasmaspheric electrons. *Geophys. Res. Lett.*, *44*(13), 6483-6491. doi: 10.1002/2017GL073885

Meredith, N. P., Thorne, R. M., Horne, R. B., Summers, D., Fraser, B. J., & Anderson,

R. R. (2003, June). Statistical analysis of relativistic electron energies for cyclotron resonance with EMIC waves observed on CRRES. *J. Geophys. Res.*, *108*, 1250-+. doi: 10.1029/2002JA009700

Ni, B., Cao, X., Zou, Z., Zhou, C., Gu, X., Bortnik, J., ... Xie, L. (2015, Sep). Resonant scattering of outer zone relativistic electrons by multiband EMIC waves and resultant electron loss time scales. *J. Geophys. Res.*, *120*(9), 7357-7373. doi: 10.1002/2015JA021466

Qin, M., Hudson, M., Li, Z., Millan, R., Shen, X., Shprits, Y., ... Kletzing, C. (2019). Investigating Loss of Relativistic Electrons Associated With EMIC Waves at Low L Values on 22 June 2015. *J. Geophys. Res.*, *124*(6), 4022-4036. doi: 10.1029/2018JA025726

Zhang, X.-J., Li, W., Thorne, R. M., Angelopoulos, V., Bortnik, J., Kletzing, C. A., ... Hospodarsky, G. B. (2016). Statistical distribution of emic wave spectra: Observations from van allen probes. *Geophys. Res. Lett.*, *43*(24), 12,348-12,355. doi: 10.1002/2016GL071158
